# Insight into Composition of Bioactive Phenolic Compounds in Leaves and Flowers of Green and Purple Basil

**DOI:** 10.3390/plants9010022

**Published:** 2019-12-23

**Authors:** Bhakti Prinsi, Silvia Morgutti, Noemi Negrini, Franco Faoro, Luca Espen

**Affiliations:** Department of Agricultural and Environmental Sciences-Production, Landscape, Agroenergy, Università degli Studi di Milano, I-20133 Milano, Italy; silvia.morgutti@unimi.it (S.M.); noemi.negrini@unimi.it (N.N.); franco.faoro@unimi.it (F.F.); luca.espen@unimi.it (L.E.)

**Keywords:** green/purple basil, LC-ESI-MS/MS, nutraceutical properties, organ chemical differences

## Abstract

Basil (*Ocimum basilicum* L.) is a culinary, medicinal, and ornamental plant appreciated for its antioxidant properties, mainly attributed to high content of rosmarinic acid. This species also includes purple varieties, characterized by the accumulation of anthocyanins in leaves and flowers. In this work, we compared the main morphological characteristics, the antioxidant capacity and the chemical composition in leaves, flowers, and corollas of green (‘Italiano Classico’) and purple (‘Red Rubin’ and ‘Dark Opal’) basil varieties. The LC-ESI-MS/MS analysis of individual compounds allowed quantifying 17 (poly)phenolic acids and 18 flavonoids, differently accumulated in leaves and flowers of the three varieties. The study revealed that in addition to rosmarinic acid, basil contains several members of the salvianolic acid family, only scarcely descripted in this species, as well as, especially in flowers, simple phenolic acids, such as 4-hydroxybenzoic acid and salvianic acid A. Moreover, the study revealed that purple leaves mainly contain highly acylated anthocyanins, while purple flowers accumulate anthocyanins with low degree of decoration. Overall, this study provides new biochemical information about the presence of not yet characterized bioactive compounds in basil that could contribute to boosting the use of this crop and to gaining new knowledge about the roles of these compounds in plant physiology.

## 1. Introduction

Basil (*Ocimum basilicum* L.) is an herbaceous plant, of the *Lamiaceae* family, traditionally cultivated worldwide and highly appreciated for its many properties. This species, which gives the common name to its whole genus, includes a large number of varieties with distinct morphological traits, chemical composition, and agro-industrial uses [1,2]. Basil includes both green varieties, with bright green leaves and white flowers, and coloured varieties, characterized by red-purple leaves and flowers, due to anthocyanin accumulation in the vacuole of epidermal cells [1,2,3]. Green basil is largely used as a culinary herb and for the extraction from leaves and flowers of aromatic essential oils, whose main components are monoterpenes and phenylpropanoids [1,2]. Purple basil encompasses varieties overall similar to green basil, such as ‘Red Rubin’, and types with peculiar traits, such as ‘Dark Opal’, that has a clove-like aroma and is used as ornamental and medicinal plant [2,4].

Several studies, mainly conducted on green varieties, showed that alcoholic and aqueous extracts from aerial parts of basil possess hypoglycemic, hepatoprotective, cardioprotective, and antimycobacterial activities, related to their antioxidant properties [5]. These properties are in part attributed to high contents of phenolic compounds, such as (poly)phenolic acids and flavonoids, which can act as reducing agents, metal chelators and free radical scavengers [6]. Rosmarinic and chicoric acids are the two most abundant (poly)phenolic acids identified in basil leaves [6,7]; for both, antioxidant and anti-viral properties were proposed [7,8]. Meanwhile, some evidence indicates that constitutive and induced accumulation of rosmarinic acid in plant leaves participates in defense against pathogens and herbivores [8].

Purple basil was also proposed as a very rich natural source of anthocyanins [3]. These compounds are the sub-class of flavonoids representing the largest group of water-soluble pigments in fruit and vegetables, and were correlated with prevention of diverse human diseases [9]. It was shown that basil has higher contents of anthocyanins than other common red fruit and medicinal herbs [3]. Moreover, purple basil contains a peculiar kind of anthocyanins, consisting of cyanidin derivatives characterized by a high degree of acylation with coumaroyl and malonyl acids [3,10]. Interestingly, it was recently demonstrated that anthocyanin extracts from purple basil leaves have anti-inflammatory in vitro activity [11]. At the same time, anthocyanins participate in several plant physiological functions, in pigmentation of flowers and seeds and in protection of vegetative organs from (a)biotic stresses [12,13]. In purple basil, anthocyanins play key roles in foliar photo-protection during stresses capable of impairing photosynthesis, such as nutrient toxicity [14] and excess solar radiation [15].

Basil varieties show high variability in the contents of total and individual phenols, as well as in antioxidant properties [16,17,18]. Despite this broad potentiality, differences among plant organs were only poorly investigated. The few studies that quantified the main phenolic compounds in leaves and flowers separately suggest distinct metabolic balances [4,16], as recently observed in *Ocimum americanum* L. [19]. However, to our knowledge, a comprehensive description of phenolic composition in flowers of green and purple varieties of the basil species is still lacking. This information could be very useful, especially considering that basil flowers are edible [2], in the light of the recent interest in the use of flowers as foods, additives or novel sources of natural compounds [20,21].

The aim of this study was therefore to compare the main plant morphological traits and the phenolic composition (contents of total phenols, antioxidant properties, and concentrations of individual (poly)phenolic acids and flavonoids) in leaves, flowers and corollas of commercial green and purple basil varieties, in order to obtain useful indications on the presence of still not characterized bioactive compounds and on their possible roles in the physiology of the plant.

## 2. Results

### 2.1. Evaluation of the Main Phenotypic Traits in Plants, Leaves and Flowers

**Figure 1:**
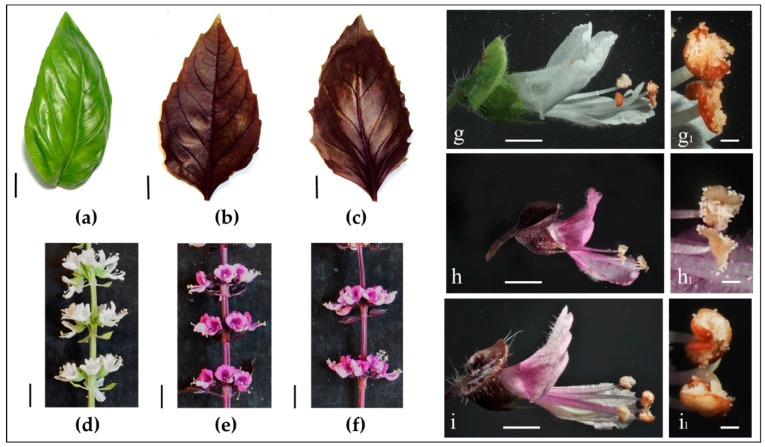
Leaves, inflorescences, and flowers of green and purple basil varieties. Left panel: (**a**) leaf of ‘Italiano Classico’; (**b**) leaf of ‘Red Rubin’; (**c**) leaf of ‘Dark Opal’; (**d**) part of inflorescence of ‘Italiano Classico’; (**e**) part of inflorescence of ‘Red Rubin’; (**f**) part of inflorescence of ‘Dark Opal’. Scale bars in black [(a)–(f)] = 1 cm. Right panel: (**g**) flower of ‘Italiano Classico’; (**h**) flower of ‘Red Rubin’; (**i**) flower of ‘Dark Opal’; anthers are enlarged in (**g_1_**), (**h_1_**), and (**i_1_**), respectively. White bars in [(g)–(i)] = 2 mm; in [(g_(1)_–(i_1_)] = 200 µm.

Basil plants of the green variety ‘Italiano Classico’ (IC) and of the purple varieties ‘Red Rubin’ (RR) and ‘Dark Opal’ (DO) were grown from seeds to full flowering in pots in greenhouse conditions. The morphological and chemical analyses of leaves were conducted at 45 days after sowing, when plants had reached maturity, but were still in the vegetative phase. At that date, IC and DO plants had similar height, of about 18 cm, while the average height of RR plants was of only about 14 cm (*p* < 0.001, Table 1). The fully expanded leaves had similar sizes (Figure 1), but they significantly differed in fresh weight, with a value of 0.53 ± 0.05 g in IC and values lower by 28% and 34% in RR and DO, respectively (*p* = 0.036 and *p* = 0.009, respectively, Table 1). The IC leaves were bright green, glossy, oval-shaped, with slightly dentate margin. RR leaves were characterized by a well evident dentate margin, while DO leaves appeared more irregularly shaped and quite undulate (Figure 1), as described in the literature [2]. The purple varieties had red-purple leaves that after visual inspection seemed a little darker in colour in DO. For both varieties, examination of hand-made transverse sections through lamina (data not shown) allowed to verify that anthocyanins were mainly located in the epidermal cells [14]. In IC and DO plants flowering started at 54 and 55 days after sowing, respectively, while RR showed a delay (*p* = 0.003 and *p* = 0.009 compared to IC and DO, respectively, Table 1). This difference is in agreement with what reported for plants grown in the field in North America [1], even if in our climate zone, in greenhouse conditions, all the three varieties started flowering earlier.

IC, RR and DO plants showed inflorescences and flowers with the typical morphology of green and purple basil [22,23]. The plants had a verticillaster type of inflorescence composed by flowers of about 1 cm in length, with a corolla formed by a larger upper lip and a smaller lower one and with declinate stamens (Figure 1). Interestingly, the fresh weight of flowers was higher in the green variety IC (*p* < 0.001), with values of 11.73 ± 0.13 mg, 9.80 ± 0.13 mg and 10.04 ± 0.22 mg in IC, RR and DO, respectively. Conversely, the weights of the corollas were comparable among the three varieties (Table 1). Together with the morphological analysis (Figure 1), this observation suggested that the differences in flower weight reside in different size and/or weight of the fertile flower parts (i.e., pistil, stamens, and pollen).

Overall, the phenotypic traits observed in both the vegetative and the reproductive stages corresponded well with the typical description of green and purple basil, confirming that the greenhouse condition allowed to maintain the varietal characteristics in IC, RR, and DO.

### 2.2. Total Phenolic Compounds, Anthocyanins Contents and Antioxidant Capacity in Leaves, Flowers and Corollas

In Table 2 are reported the contents in total phenolic compounds and anthocyanins, as well as the antioxidant capacity evaluated in leaves, flowers, and corollas of the green variety IC and of the two purple varieties RR and DO.

The contents of total phenolic compounds were different comparing the same organ among the three varieties (Table 2, D_var_), as well as the different organs within each variety (Table 2, D_org_). The contents of total phenolic compounds were always higher in the leaves (*p* < 0.001), followed by the values of flowers and corollas. In leaves, the highest value of 7.11 ± 0.14 mg GAE g^−1^ FW was measured in RR (*p* < 0.001 and *p* = 0.005 compared to IC and DO, respectively), while IC and DO showed lower and comparable levels. On the contrary, the contents of total phenols in flowers and corollas were similar in RR and DO, and in all the comparisons they were higher than the values determined in IC (*p* = 0.038, *p* = 0.010 and *p* = 0.006, *p* < 0.001 in flowers and corollas, respectively).

The evaluation of anthocyanin contents did not reveal any significant difference between RR and DO. In both the purple varieties, antocyanins were accumulated to a higher extent in the leaves (*p* < 0.001), while they were present at lower and similar levels in flowers and corollas. This evidence suggests that also the fertile parts of the flower contain anthocyanins, as supported by the observation that in these two varieties the stamens were also coloured (Figure 1). As expected, the anthocyanin contents observed in IC were always very low.

The antioxidant capacity followed a peculiar trend (Table 2). In leaf extracts, this parameter did not reveal any significant difference between green and purple varieties, showing values very similar in IC, RR, and DO. Moreover, in all the three varieties, flower and corolla extracts showed significantly higher antioxidant capacity (*p* < 0.001), with about a 2-fold increase respect to the leaf extracts. Interestingly, white flowers showed a significantly higher antioxidant capacity (*p* = 0.023 and *p* < 0.001 compared to RR and DO) than the purple ones, despite the minor content of phenols and anthocyanins. Finally, the analysis showed similar values of polyphenols and anthocyanins in the two purple flowers and corollas, whereas the antioxidant capacity was higher in RR (*p* < 0.001).

Overall, the patterns observed for the three parameters suggested that the differences in antioxidant capacity among organs and varieties could be ascribed not directly to the total contents of phenolic compounds (and anthocyanins), but more probably to different specific compositions of these classes of compounds.

### 2.3. Identification and Quantification of Individual (Poly)phenolic Acids

**Figure 2:**
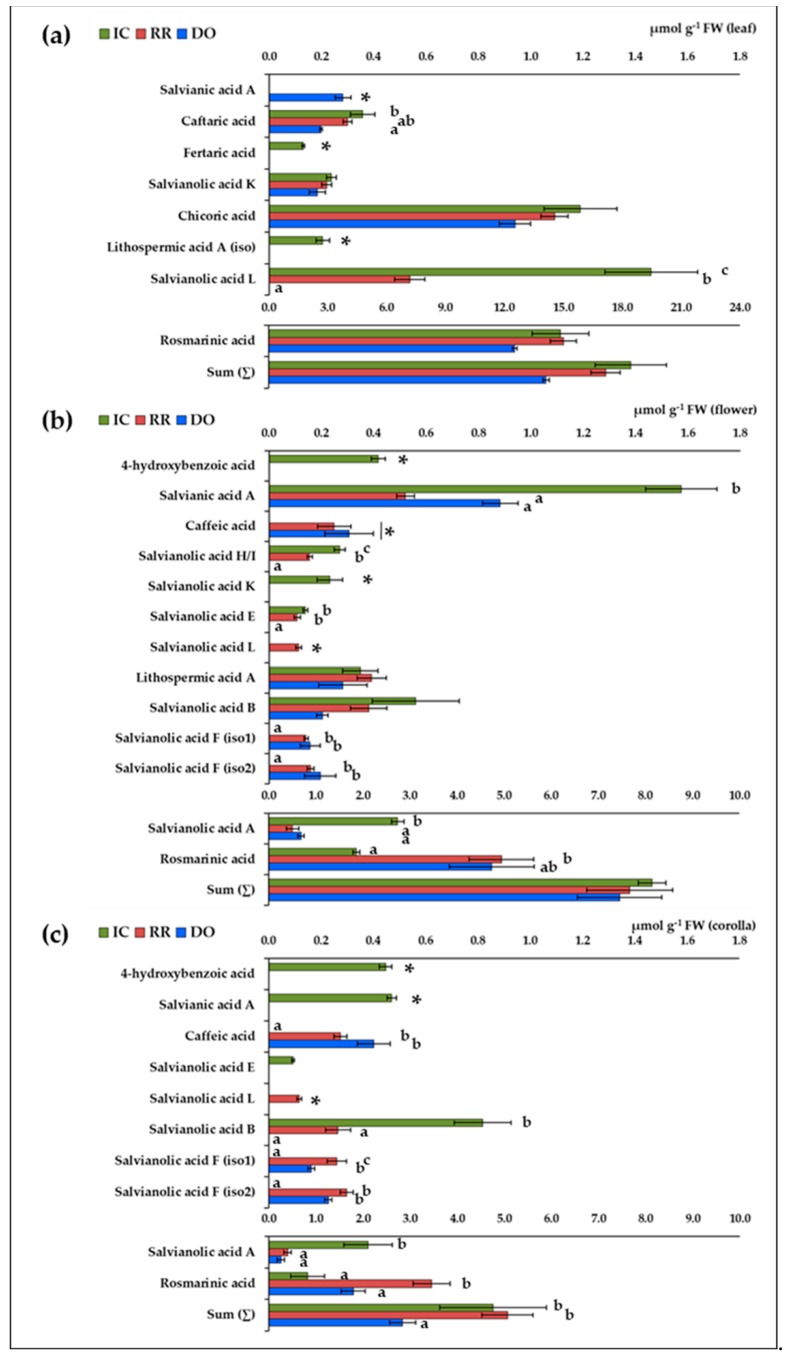
Contents of (poly)phenolic acids in green and purple basil. (**a**) leaf; (**b**) flower; (**c**) corolla. Values are the means ± SE (error bars; *n* = 3) expressed as µmol g^−1^ FW. Data are grouped in two scales to visualize differences. Significant differences were assessed by one-way ANOVA test (*p* ≤ 0.05, Tukey post hoc); (*) significant difference respect to zero assessed by one-Sample t-test (*p* ≤ 0.05).

The quantification of individual (poly)phenolic acids in green and purple basil varieties was conducted by LC-ESI-MS/MS (liquid-chromatography tandem mass spectrometry). This approach allowed to characterize 17 compounds, identified on the basis of their molecular weight and fragmentation profile, according to the literature (Table 3). The concentrations of these (poly)phenolic acids in leaves, flowers and corollas in IC, RR and DO are reported in Figure 2.

If needed, in order to tentatively achieve selection of different isomers, the compounds were assigned by comparing different information. In detail, 4-hydroxybenzoic acid and salicylic acid give identical fragmentation profiles, but it was possible to assign the chromatographic peak to 4-hydroxybenzoic acid (n.1) since it elutes at an earlier retention time respect to a standard of salicylic acid, as reported by literature [24]. Salvianic acid A (n.2) [25] was discriminated from its isomers syringic acid and ethyl gallate on the basis of different fragmentation profiles [24]. Fertaric acid (n.5) was identified by comparing our results to the fragmentation profile proposed by Lee and Scagel in basil leaf [7], i.e., 325 → 282, 193, 149 *m*/*z*, and to that proposed by Khoza et al. [26], i.e., 325 → 193, 149, 134 *m*/*z*. Most likely, these differences were due to a different collision energy during the MS/MS experiments. As far as it regards the isomers of lithospermic acid A (n.6,9,14), it was possible to identify the salvianolic acid H/I (n.6) by the presence of the base peak at 339 (100) *m*/*z*, according to Ruan et al. [27], while lithospermic acid A (n.14) and its isomers (n.9) were tentatively assigned on the basis of RT and proportion among fragmentation peaks [i.e., 359 (99) and 259 (100) *m*/*z*, respectively], as proposed by Barros et al. [29]. Salvianolic acid K was tentatively assigned according to the nomenclature proposed in *Salvia officinalis* L. [28] and to the fragmentation similitude to the other lithospermic acid derivatives. On the other hand, the profiles obtained for the isomers of salvianolic acid B (n.10,11,15), even if consistent with the literature [27,29], did not provide adequate information for their discrimination. Salvianolic acid B (n.15) was identified by comparison to the standard molecule, but salvianolic acid E (n.10) and L (n.11) were only tentatively assigned according to RT [27].

The evaluation of the concentrations of individual (poly)phenolic acids showed that rosmarinic acid was the compound accumulated at the highest amounts in leaves, accounting for more than 80% of the sum of all the molecules detected, followed by chicoric and caftaric acids (Figure 2a). These molecules were accumulated to similar levels in the three basil varieties, with the exception of the higher concentration of caftaric acid in the IC leaves than in the DO ones (*p* = 0.019). Interestingly, the analysis revealed the presence of other (poly)phenolic acids. Aside from comparable amounts of salvianolic acid K, these compounds were differently accumulated in the three varieties. IC leaves were characterized by the accumulation of fertaric acid and lithospermic acid A (iso), as well as by the highest level of salvianolic acid L (*p* = 0.002 and *p* < 0.001 compared to RR and DO, respectively). In particular, salvianolic acid L level was less than half in RR and undetectable in DO leaves. The lower accumulation of these compounds in DO seemed partially balanced by the peculiar accumulation of salvianic acid A (Figure 2a). Although the sum of all the (poly)phenolic acids in intact flowers resulted similar in the three basil varieties, the analysis revealed a complex and various composition, embracing several members of the salvianolic acid family (Figure 2b). Once again, rosmarinic acid was one of the more abundant (poly)phenolic acids, but it resulted accumulated to higher levels in the purple flowers than in the white ones (*p* = 0.044, comparing IC and RR). Interestingly, an opposite trend was observed for salvianolic acid A that reached very high levels in IC flowers (*p* = 0.005 and *p* = 0.008 compared to RR and DO, respectively). In addition, IC flowers were characterized by a peculiar accumulation of 4-hydroxybenzoic acid and salvianolic acid K, as well as by significantly higher levels of salvianic acid A (*p* < 0.001 and *p* = 0.004 compared to RR and DO, respectively) and salvianolic acid H/I (*p* = 0.002 and *p* < 0.001 compared to RR and DO, respectively). The analysis did not reveal differences in the concentrations of lithospermic acid A and salvianolic acid B, but it showed a different composition for salvianolic acids E and L. Finally, purple flowers of RR and DO were characterized by similar contents of caffeic acid and salvianolic acid F isomers, not detected in white flowers. Overall, the total amounts of poly(phenolic) acids in isolated corollas was lower than in flowers (*p* = 0.001), especially in DO, where it reached a minimum value, mainly due to lower accumulation of rosmarinic acid (*p* = 0.021, Figure 2c). In IC the low level of rosmarinic acid seemed partially compensated by salvianolic acid A (*p* < 0.001), 4-hydroxybenzoic acid (*p* = 0.003), salvianic acid A (*p* = 0.002), and also by the highest level of salvianolic acid B (*p* = 0.003 and *p* < 0.001) compared to RR and DO. The corolla composition resulted quite comparable to that of the respective intact flowers in all the three basil varieties. A very interesting observation was that lithospermic acid A, salvianolic acids H/I and K were not detected in isolated corollas, and the concentrations of salvianic A were significantly lower in corollas than in flowers (*p* < 0.001), suggesting that these molecules are preferentially accumulated in the fertile parts of the flowers.

### 2.4. Identification and Quantification of Individual Flavonoids

**Figure 3:**
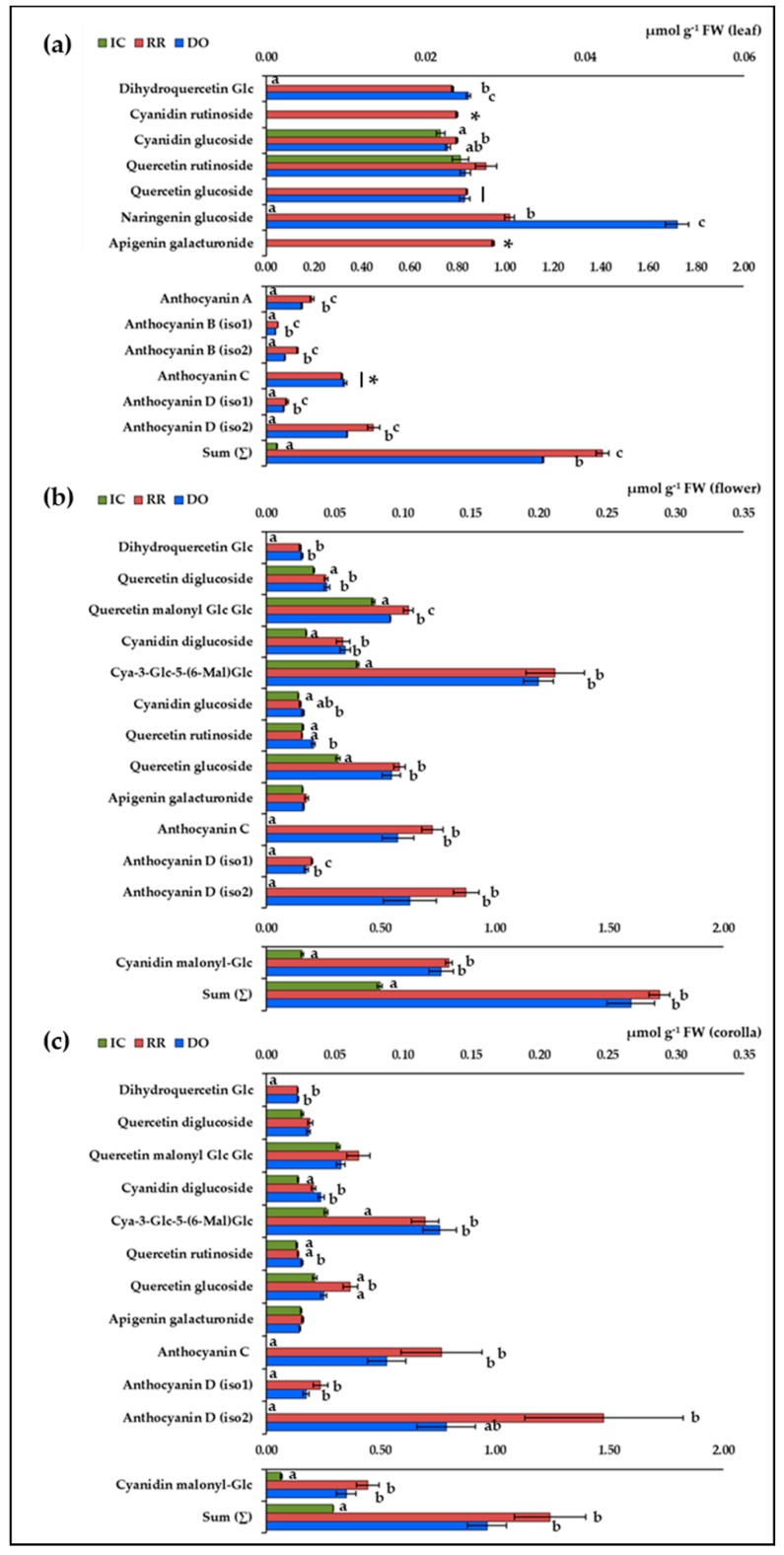
Contents of flavonoids in green and purple basil. (**a**) leaf; (**b**) flower; (**c**) corolla. Values are the means ± SE (error bars; *n* = 3) expressed as µmol g^−1^ FW. Data are grouped in two scales to visualize differences. Significant differences were assessed by one-way ANOVA test (*p* ≤ 0.05, Tukey post hoc); (*) significant difference respect to zero assessed by one-Sample t-test (*p* ≤ 0.05).

The quantification of individual flavonoids in green and purple basil varieties was conducted by LC-ESI-MS/MS in positive mode. This approach allowed to tentatively identify 18 compounds on the basis of their molecular weight and fragmentation profiles, according to the literature (Table 4). The concentrations of these flavonoids in leaves, flowers and corollas in IC, RR and DO are reported in Figure 3.

The LC-ESI-MS/MS analysis confirmed that the major flavonoids accumulated in basil are cyanidin derivatives, accounting for 11 molecules out of the total 18 flavonoids identified. These molecules were identified on the basis of the presence in their fragmentation profile of the ions at 287.06 *m*/*z*, corresponding to the aglycon moiety. As regards anthocyanins with higher molecular weight (n.23,27,28,29,30,33,35), our results confirmed the description recently provided by Luna et al. [10] by an MS^2^-MS^3^ approach. Considering that our approach was based only on MS^2^ spectrum profiles, the molecule assignments matched with a very high reliability, and were also coherent on the basis of RT. Although we verified the chemical information reported in the cited study, whenever possible we adopted, for the sake of clarity, the simpler nomenclature proposed by Flanigan and Niemeyer [18]. In detail, Cya-3-Glc-5-(6-Mal)Glc (n.23) lacked the ion at 535.11 *m*/*z*, but the presence of the ion at 449.11 *m*/*z*, following the rule of the preferential fragmentation through the hydroxyl group at position 5 (-248 *m*/*z* of Mal-Glc moiety) [10], confirmed the glycosidic fractions on positions 5 and 3. For anthocyanin B (1167.28 *m*/*z*) and D (1151.29 *m*/*z*), the results confirmed the presence of two isomers, differently substituted. The couple of ions at 919.23/535.11 *m*/*z* and at 903.23/535.11 *m*/*z* in the profile of the isomers B1 (n.28) and D1 (n.33) revealed the presence of a malonyl-glucoside moiety at position 5. Meanwhile, the higher intensity of the couple of ions at 1005.23/449.11 *m*/*z* and at 989.24/449.11 *m*/*z* indicated a simple glucosylation at 5 in the isomers B2 (n.29) and D2 (n.35), respectively.

Although these results confirmed those obtained by Luna et al. [10] in ‘Purple Iranian Basil’, our analysis did not detect the described presence of two other isomers of anthocyanin B, as well as of other six less abundant anthocyanins. In our opinion, this discrepancy derived from the fact that, interestingly, the major highly glycosylated anthocyanins (and isomers) showed different quantitative proportions among the three purple varieties. However, in addition our analysis allowed detecting, by comparison to standard or to previous literature [10,30], anthocyanins with lower molecular weight (n.21,22,24,34).

Moreover, it was possible to identify other classes of flavonoids. Quercetin derivatives (n.19,20,25,26) were assigned by combining information related to the *m*/*z* of the precursor with the presence of the ion at 303.05 *m*/*z* (quercetin aglycon) in the fragmentation profile [30,32]. In the case of quercetin malonyl Glc Glc (n.20), loss of the malonylglucoside moiety (713.16–248.05 → 465.11, corresponding to quercetin glucoside) was also detected. Dihydroquercetin glucoside (n.18) was assigned on the basis of the matching between the precursor (467.12 *m*/*z*) and the fragmentation of the aglycon proposed by Abad-García et al. [30,31]. Similarly, naringenin glucoside (n.31) and apigenin galacturonide (n.32) were identified by the ion of the aglycon (273.08 and 271.06 *m*/*z*, respectively) and, for the first one, by the relative fragmentation described in the literature [32,33].

The quantification of flavonoids in leaves of IC, RR, and DO (Figure 3a) confirmed that the highly acylated anthocyanins are the predominant molecules [3,10,18]. Although colorimetric analysis did not detect differences in the contents of total anthocyanins in RR and DO leaves, the MS^2^ approach revealed that these molecules were more abundant in RR than in DO, with the exception of anthocyanin C. As far as it concerns the minor flavonoids, the more evident differences resided in the higher accumulation of dihydroquercetin glucoside and naringenin glucoside in DO compared to RR (*p* < 0.001), and in the peculiar accumulation of cyanidin rutinoside and apigenin galacturonide in RR leaves. Finally, it was possible to observe that cyanidin glucoside and quercetin rutinoside were also accumulated in the leaves of the green variety.

The analysis revealed a different composition in intact flowers (Figure 3b). Anthocyanins A and B were not detected, and anthocyanins C and D were only minor compounds. This seemed to be partially compensated by the accumulation of low acylated anthocyanins (i.e., cyanidin-3-Glc-5-(6-Mal)Glc and cyanidin malonyl-glucoside). Moreover, it was observed a higher concentration of cyanidin derivatives with low molecular weight as well as of quercetin derivatives, some of which were also accumulated in white flowers. Differently than in leaves, the flavonoid composition in flowers resulted very similar in the two purple varieties. Finally, analysis of isolated corollas revealed concentrations and compositions of flavonoids similar to those observed in intact flowers (Figure 3).

## 3. Discussion

The analysis of phenotypic traits in ‘Italiano Classico’ (IC), ‘Red Rubin’ (RR) and ‘Dark Opal’ (DO) plants allowed to highlight interesting differences between green and purple basil varieties in both vegetative and reproductive stages. As far as it regards leaves, the choice to perform the study during vegetative growth derived from the fact that this phase corresponds to the main harvest period for basil crops in the Mediterranean area [2]. Since the accumulation of anthocyanins in basil increases during growth, but declines after flowering [3], the choice to conduct the study in this developmental phase was intended to reduce the possible loss of phenolic compounds. The higher leaf fresh weight in IC (Table 1) suggests anatomical differences and is in agreement with the observation that the green variety ‘Tigullio’ tends to have leaves thicker and with more compact mesophyll than RR [15]. The higher fresh weight of flowers in IC (Table 1) could be related to different factors. On one hand, this feature could be due to a higher production of pollen. In 2007 Chwil [34] indeed reported higher nectar weight and bigger pollen grains in a green variety compared to a purple one. On the other hand, this difference could also be related to a prolonged juvenility in white flowers as compared to coloured ones, as described in *Petunia × hybrida* [30]. Since basil is a melliferous species of great value [23,34], future studies aimed to clarify this aspect could have important implications.

As far as it regards the contents of total phenols in basil, it is important to notice that the values reported in the literature differ of up to one order of magnitude. This variability could be probably due to differences in the harvest time of plants, in the use of fresh or dried tissues for the analytical determinations, and in the methodological approaches adopted. The concentrations of total phenols in leaves, evaluated in dried tissues of several basil cultivars, ranges from 5 to 27 mg GAE g^−1^ DW [17,18], lower than the values of 5–7 mg GAE g^−1^ FW observed in the present work (Table 2). However, our results are consistent with those of Lee and Scagel [7] that report values of about 6 mg GAE g^−1^ FW in the green variety ‘Genovese Italian’. Concerning anthocyanins, whether determined by HPLC [3,10] or colorimetric [17,18] methods, their total concentrations in purple basil leaves range from 0.06 to 0.55 mg CGE g^−1^ FW or from 0.8 to 1.6 mg CGE g^−1^ FW, respectively (similar to our results).

The present study provides novel information, especially about flowers. Our results confirm that phenols are accumulated in flowers, as previously described in ‘Dark Opal’ [4] and in Iranian basil accessions [16], and suggest that the quantitative differences between white and purple flowers could be mainly ascribed to anthocyanins (Table 2). Moreover, our work confirms a high similarity in anthocyanin concentrations in RR and DO, as described in the entire aerial part of plants at flowering [3], and also provides additional details about the characteristics of leaves, flowers, and corollas. Finally, the results allow highlighting that in all the studied varieties the fertile parts of flowers accumulate significant amounts of phenols that may play specific, even if not yet elucidated, physiological roles in such reproductive tissues.

This work also provides novel information about the antioxidant capacity in the different organs of basil varieties; this property shows only a weak relationship with the total phenols and anthocyanin contents, as previously discussed [4,17,18], suggesting that the antioxidant activity depends on specific molecules. Moreover, the results show that flowers, and in particular the white ones, possess a higher antioxidant capacity than leaves (Table 2). This is consistent with what was observed in *O. americanum* [19] but not in *O. sanctus* [35], suggesting a different chemical composition in flowers of the different *Ocimum* species.

The above detailed considerations prompted us to investigate the composition, in the different organs and basil varieties, of individual (poly)phenolic acids and flavonoids.

The analysis of the concentrations of individual (poly)phenolic acids in IC, RR and DO varieties confirms that rosmarinic and chicoric acids are the predominant polyphenolic acids in basil leaves [6,7]. The fact that these molecules were accumulated at similar amounts in all the three varieties is probably one of the major factors determining the similar antioxidant capacity of the leaves (Figure 2, Table 2). Overall, these results are consistent with data from the literature reporting comparable levels of rosmarinic and chicoric acids in purple and green basil [17], and in different purple varieties [18]. At the same time, our study confirms that rosmarinic acid is also accumulated in flowers [4,16], but reveals the absence of chicoric acid. This biochemical difference between organs could be a useful tool in future studies to gain new information about chicoric acid biosynthesis, presently not yet completely elucidated [36].

The results show that basil also accumulates simple phenolic acids and several members of the salvianolic acid family (Figure 2). The presence of lithospermic acid A in leaves [7] as well as that of salvianolic acid B, caffeic acid, and 4-hydroxbenzoic acid in flowers [16] has been reported in recent years, but only marginally investigated. Caffeic acid and salvianic acid A are the structural units of the salvianolic acids [37]. In particular, salvianic acid A is considered a superior antioxidant and one of the main therapeutic components for cardiac dysfunctions [25,38]. Moreover, it was proposed that salvianolic acids, identified as the main bioactive compounds in *Salvia* spp., could possess pharmacological properties useful to counteract a wide range of human pathologies [37,39]. The presence of these compounds in basil could improve the nutraceutical and technological value of this crop for food and pharmaceutical industry. Indeed, considering that the nutraceutical benefits of vegetables are ever more associated with additive and synergistic combinations of phytochemicals [40], it is conceivable that salvianic acid and salvianolic acids, even if present at low amounts, could contribute to the beneficial properties of basil. This consideration could be of particular importance for flowers, where the bulk of these phenolic acids reached a concentration similar to that of rosmarinic acid. These (poly)phenolic acids resulted accumulated to a higher extent in IC flowers, suggesting that these molecules may contribute to the highest antioxidant properties of white flowers. Absence of salvianolic acids H/I, K, E, and L could account for the lowest values of antioxidant capacity observed in DO flowers (Figure 2, Table 2). This trend is in agreement with a recent study that compares white and purple flowers of *Magnolia* spp., where the antioxidant capacity was related to the levels of salvianic acid A [41]. It is also noteworthy that flower composition differed in the analyzed varieties, especially as far as it concerns the simpler phenolic acids. In particular, 4-hydroxybenzoic acid and salvianolic acid K were specific of IC, where in addition salvianic acid A and salvianolic acid A were accumulated to higher extents, while caffeic acid and salvianolic acid F were specific of purple flowers (Figure 2). Considering that in *Elsholtzia rugulosa* (Hemsl.; *Lamiaceae*) flowers the accumulation of 4-hydroxybenzoic acid is a key factor in the selective attraction of pollinators [42], it is possible that in basil flowers phenolic acids contribute in diversifying the interactions between a specific variety and insects. Finally, the higher levels of salvianic acid A, salvianolic acids H/I and K, and lithospermic acid A in flowers than in corollas suggest that these compounds are more accumulated in the fertile parts of flowers (Figure 2). This consideration supports the hypothesis that (poly)phenolic acids could have, for plants, other physiological roles, besides their antioxidant properties.

As far as it concerns the analysis of individual flavonoids in leaves and flowers of IC, RR, and DO varieties, one of the more interesting aspects is the observation that highly acylated anthocyanins typically characterizing purple basil (i.e., anthocyanins A–D and their isomers) [3,10,18] are effectively predominant only in leaves, whereas flowers accumulate anthocyanins with lower degree of decoration (Figure 3). This observation has several implications. From a methodological point of view, it is important to notice that in comparison to the colorimetric evaluation, the HPLC-MS/MS approach seems to significantly underestimate the anthocyanin concentration in leaves (Table 2, Figure 3). As previously stated [3,10], it is possible that the lack of adequate standards affects the MS analysis, bringing to underestimation of the highly acylated anthocyanins, probably due to different ionization efficiency. In our opinion, this aspect should be taken into account in the critical evaluation of the literature, as well as in the optimization of analytical approaches. The different anthocyanin composition in leaves and flowers could also affect their technological properties, especially because the degree of acylation and glucosylation affects their stability, chemical reactivity, and antioxidant properties [9]. Finally, this difference could be related to physiological aspects. Indeed, it was observed that at similar concentration, acylated anthocyanins absorb significantly more visible and UV-B light than the simpler anthocyanin equivalents [43]. Considering that, in purple basil, anthocyanins play key roles in photo-protection [14,15], it is possible to hypothesize that leaves have evolved the metabolic ability to accumulate a selected set of anthocyanins specifically involved in this process. In this regard, it is important to stress that the accumulation of highly acylated anthocyanins has a high metabolic cost, especially in terms of C skeletons, and hence it is conceivable that this ability is adequately sustained in photosynthetic tissues, but it declines in heterotrophic floral tissues. From a biochemical point of view, this work appears to provide the basis for future studies aimed at clarifying the biochemical mechanisms involved in the regulation of the different anthocyanin composition in leaves and flowers of purple basil varieties.

In conclusion, the biochemical picture of the green and purple basil varieties concerning their phenolic components revealed the presence of bioactive compounds not yet characterized in this species, while also providing new clues about the nutraceutical properties as well as the possible roles of these compounds in the physiology of this plant.

## 4. Materials and Methods

### 4.1. Plant Material and Growing Conditions

Basil seeds (*O. basilicum* L.) of the selected varieties ‘Italiano Classico’ (IC), ‘Red Rubin’ (RR) and ‘Dark Opal’ (DO) were purchased from Franchi Sementi (Grassobbio, Italy). Plants were grown individually in 5-l pots filled with commercial universal soil (neutral peat and expanded perlite < 5%, pH 6.8, with 0.5 kg m^−3^ of slow-release fertilizer (NPKS 14/7/17/9 plus B 0.02 and Zn 0.01) and added with 1.25 g/L of organic mineral fertilizer (NKMgS 5/8/3/16)) and watered daily with tap water. The experiment was conducted between March and July 2018, in a greenhouse in Milan (Italy) implemented with supplementary light and a cooling system (16 h/8 light-dark photoperiod, PPFD of about 600 μmol of photons m^−2^ s^−1^, 20–25 °C night/day), randomizing pot arrangement of the three varieties.

Forty-five days after sowing the vegetative phenotypic parameters were recorded and leaf samples were collected, selecting six fully expanded leaves from a total of three plants, for each biological replicate. Flowers were collected within two days after anthesis, choosing the ones showing lack of damage or browning. Individual flower samples were obtained after removal of pedicel and sepals. Corollas were obtained removing pistil and stamens by means of blunt tweezers under a magnifying glass. Flower and corolla samples were composed of 18 flowers collected from three inflorescences. All samples were weighed, immediately frozen in liquid N_2,_ and stored at −80 °C for further use. The images of flowers and anthers were obtained by a stereo microscope Axio Zoom Zeiss V16, Carl Zeiss A.G., Feldbach, Switzerland.

### 4.2. Determination of the Total Phenol Compounds and Anthocyanins Contents

Frozen samples (150 mg) were finely powdered (mortar and pestle) in liquid N_2_, extracted by gentle shaking in 3 volumes of methanol, and centrifuged at 14,000 g for 10 min at 4 °C. Samples were diluted in 1:6 (*v*:*v*) ratio with 70% (*v*:*v*) methanol. The concentration of total phenolic compounds was determined by the microscale Folin–Ciocalteau colorimetric method [44]. The absorbance at 765 nm was referred to a calibration curve of gallic acid (GA) and expressed as mg gallic acid equivalents (GAE) g^−1^ fresh weight (FW). The antioxidant capacity was determined by a colorimetric method based on the reduction of Mo(VI) and formation of a green phosphate/Mo(V) complex at acidic pH [45]. The absorbance at 695 nm was referred to a calibration curve of ascorbic acid (AA), and expressed as µmol AA equivalents (AAE) g^−1^ FW.

Total anthocyanin concentration was quantified in the same extracts used for the analysis of individual anthocyanins. After dilution of the samples from purple tissues in a 1:5 ratio in acidified methanol (50% (*v*:*v*) methanol, 0.5% (*v*:*v*) formic acid (FA)), the absorbance at 520 nm was referred to a calibration curve of cyanidin-3-O-glucoside (CG) [46]. The concentrations of anthocyanins in the samples were reported as µmol CG equivalents (CGE) g^−1^ FW. All the analyses were conducted in three biological samples (*n* = 3).

### 4.3. Quantification of Individual (Poly)Phenolic Acids and Flavonoids

The analysis of individual (poly)phenolic acids and flavonoids was conducted on the same plant extracts, obtained as previously described by Tattini and coworkers [15], with minor modifications. Briefly, frozen samples of leaves, flowers and corollas were finely powdered in liquid N_2_ by mortar and pestle. Samples (100 mg) were extracted in 6 ml of CHCl_3_:CH_3_OH:H_2_O (12:5:1, pH 2 by addition of FA) by shaking at 4 °C for 30 min, followed by mild sonication at 4 °C for 5 min. The samples were then added with 3.32 ml of CHCl_3_ plus 1.54 mL of 0.5% (*v*:*v*) FA, shaken at 4° C for 20 min, and centrifuged at 3000*g* for 10 min. The upper phase was collected, filtered by sterilized polyvinylidene difluoride (PVDF) membrane (0.45 µm; Waters S.p.A., Sesto San Giovanni, Italy). After dilution, samples were analyzed by capillary HPLC (Agilent Technologies 1200 series) coupled with ESI source on 6520 Q-TOF mass spectrometer (Agilent Technologies Italia S.p.A, Cernusco s/N, Italy). LC runs were performed on a XDB-C18 column (2.1 × 50 mm, 1.8 µm) in acidic condition [0.1% (*v*:*v*) FA] applying the following acidified acetonitrile (0.1% FA) gradient: 0–2 min at 5%, 2–15 min to 15%, 15–45 min to 45%, with a flow rate of 200 µL min^−1^. The analysis of (poly)phenolic acid compounds was conducted in negative mode, setting the ESI source at 350 °C and −3000 V. The analysis of flavonoids was conducted in positive mode, setting the ESI source at 350 °C and +3500 V. In both modes, data acquisition was in a range of 100–1500 *m*/*z*. The targeted MS/MS analyses were performed applying a collision energy of 15 V and 25 V in negative and positive modes, respectively. The quantification of compounds was done on MS spectra by extracting the EIC (extracted ion current) with tolerance of 40 ppm, using adequate external calibration curves selected on the basis of molecular weight and chemical similitude. In detail, caffeic acid was used to calibrate itself, 4-hydroxybenzoic acid and salvianic acid A; rosmarinic acid was used to calibrate itself, caftaric acid, fertaric acid, salvianolic acid F; chicoric acid was used to calibrate itself, salvianolic acids A, I, K and lithospermic acid; salvianolic acid B was used to calibrate itself and its isomers; cyanidin rutinoside was used as a standard for flavonoids. Quantitative analyses were conducted on three biological replicates (*n* = 3), verifying molecule assignments by MS/MS analysis in each condition.

## Figures and Tables

**Table 1:** Evaluation of phenotypic traits in green and purple basil varieties (*O. basilicum* L.). The plant height and the fresh weight of fully expanded leaves were evaluated at 45 days after sowing. Values are means ± SE (*n* = 6; ^1^ *n* = 12). Statistically significant differences were assessed by one-way ANOVA test (*p* ≤ 0.05, Tukey post hoc, letters in brackets within each row).

Plant Characteristic	Variety		
	‘Italiano Classico’	‘Red Rubin’	‘Dark Opal’
Plant height ^1^ (cm)	18.83 ± 0.58 (b)	13.58 ± 0.47 (a)	18.00 ± 0.39 (b)
Leaf fresh weight (g)	0.53 ± 0.05 (b)	0.38 ± 0.03 (a)	0.35 ± 0.03 (a)
Days to flowering	54 ± 1 (a)	59 ± 1 (b)	55 ± 1 (a)
Flower fresh weight (mg)	11.73 ± 0.13 (b)	9.80 ± 0.13 (a)	10.04 ± 0.22 (a)
Corolla fresh weight (mg)	7.94 ± 0.17 (a)	7.51 ± 0.16 (a)	7.04 ± 0.44 (a)

**Table 2:** Total phenolic compounds and anthocyanins contents and antioxidant capacity in leaves, flowers, and corollas of green and purple basil (*O. basilicum* L.) varieties. IC: ‘Italiano Classico’; RR: ‘Red Rubin’; DO: ‘Dark Opal’; GAE: gallic acid equivalents; CGE: cyanidin-3-O-glucoside equivalents; AAE: ascorbic acid equivalents. Values are means ± SE (*n* = 3). Statistically significant differences were assessed by two-way ANOVA test (*p* ≤ 0.05, Tukey post hoc); D_var_: significant differences among varieties within each organ (leaf: lower case, flower: upper case, corolla: italic); D_org_: significant differences among organs within each variety (IC: lower case, RR: upper case, DO: italic).

Organ	Variety	Total Phenols (mg GAE g^−1^ FW)	D_var_	D_org_	Anthocyanins (µmol CGE g^−1^ FW)	D_var_	D_org_	Antioxidant Capacity (µmol AAE g^−1^ FW)	D_var_	D_org_
Leaf	IC	5.57 ± 0.29	a	c	0.01 ± 0.01	a	a	63.48 ± 2.00	a	a
	RR	7.11 ± 0.14	b	C	3.96 ± 0.05	b	B	68.98 ± 2.12	a	A
	DO	6.07 ± 0.22	a	*c*	3.68 ± 0.20	b	*b*	61.45 ± 0.62	a	*a*
Flower	IC	3.58 ± 0.06	A	b	0.02 ± 0.01	A	a	148.18 ± 2.51	C	b
	RR	4.35 ± 0.10	B	B	0.87 ± 0.11	B	A	133.45 ± 7.86	B	B
	DO	4.54 ± 0.33	B	*b*	0.75 ± 0.12	B	*a*	106.44 ± 4.45	A	*b*
Corolla	IC	2.30 ± 0.16	*a*	a	0.03 ± 0.02	*a*	a	147.59 ± 1.23	*b*	b
	RR	3.32 ± 0.03	*b*	A	1.30 ± 0.27	*b*	A	135.41 ± 2.44	*b*	B
	DO	3.74 ± 0.25	*b*	*a*	1.00 ± 0.14	*b*	*a*	99.96 ± 3.00	*a*	*b*

**Table 3:** (Poly)phenolic acids identified in the green and purple basil varieties by LC-ESI-MS/MS. n: number of peak. RT: retention time. [M−H]^−^: molecular ion detected in negative mode (*m*/*z*: mass/charge). Ref.: reference. (iso): isomer. The RT and fragmentation profile of each compound was verified in all varieties and organs. Quantification of individual compounds is reported in Figure 2.

n.	Compound	RT (min)	Formula	[M−H]^−^(*m*/*z*)	MS^2^ Fragmentation Profile (*m*/*z*) ^1^	Ref.
1	4-hydroxybenzoic acid	1.5	C_7_H_6_O_3_	137.02	137.02 (5), 93.03 (100) ^2^	[24]
2	Salvianic acid A	2.5	C_9_H_10_O_5_	197.04	197.04 (14), 179.03 (70), 135.04 (84), 123.04 (57), 72.99 (100)	[24,25]
3	Caftaric acid	5.3	C_13_H_12_O_9_	311.04	179.03 (100), 149.01 (79), 135.04 (14)	[26]
4	Caffeic acid	8.6	C_9_H_8_O_4_	179.03	179.03 (25), 135.04 (100)	[27] ^2^
5	Fertaric acid	9.0	C_14_H_14_O_9_	325.06	193.05 (100), 134.04 (12)	[7,26]
6	Salvianolic acid H/I	11.3	C_27_H_22_O_12_	537.10	537.10 (5), 493.11 (66), 339.05 (100), 313.07 (8), 295.06 (34), 197.04 (27), 179.03 (8)	[27]
7	Salvianolic acid K	11.7	C_27_H_24_O_13_	555.11	537.10 (10), 493.11 (56), 295.06 (100)	[28,29] ^3^
8	Chicoric acid	12.0	C_22_H_18_O_12_	473.07	311.04 (100), 293.03 (24), 179.03 (48), 149.01 (82)	[26] ^2^
9	Lithospermic acid A (iso)	12.3	C_27_H_22_O_12_	537.10	537.10 (48), 493.11 (77), 295.06 (100)	[29] ^3^
10	Salvianolic acid E	13.9	C_36_H_30_O_16_	717.15	717.15 (100), 519.09 (73), 475.10 (19), 339.05 (7)	[27,29] ^3^
11	Salvianolic acid L	14.8	C_36_H_30_O_16_	717.15	717.15 (68), 673.16 (10), 537.10 (26), 519.09 (71), 321.04 (5), 295.06 (5)	[27,29] ^3^
12	Rosmarinic acid	15.2	C_18_H_16_O_8_	359.08	359.08 (13), 197.04 (35), 179.03 (8), 161.02 (100)	[27] ^2^
13	Salvianolic acid A	15.6	C_26_H_22_O_10_	493.11	493.11 (21), 313.07 (7), 295.06 (100), 185.02 (17)	[27]
14	Lithospermic acid A	16.7	C_27_H_22_O_12_	537.10	493.11 (99), 359.08 (99), 313.07 (13), 295.06 (28), 197.04 (9), 179.03 (14), 161.02 (29), 135.04 (9)	[29] ^3^
15	Salvianolic acid B	17.4	C_36_H_30_O_16_	717.15	717.15 (23), 519.09 (100), 321.04 (10)	[27] ^2^
16	Salvianolic acid F (iso1)	22.0	C_17_H_14_O_6_	313.07	161.02 (100)	[29] ^3^
17	Salvianolic acid F (iso2)	23.7	C_17_H_14_O_6_	313.10	161.02 (100)	[29] ^3^

^1^ In brackets are reported the average relative abundances of each fragment ion. ^2^ Identified by standard. ^3^ Tentatively assigned.

**Table 4:** Flavonoids identified in the green and purple basil varieties by LC-ESI-MS/MS. n: number of peak. RT: retention time. [M+H]^+^: molecular ion detected in positive mode (*m*/*z*: mass/charge). Ref.: reference. (iso): isomer. Glc: glucoside. Mal: malonyl. The RT and fragmentation profile of each compound was verified in all varieties and organs. Quantification of compounds is in Figure 3.

n.	Compound	RT (min)	Formula	[M+H]^+^ (*m*/*z*)	MS^2^ Fragmentation Profile (*m*/*z*) ^1^	Ref.
18	Dihydroquercetin glucoside	7.9	C_21_H_22_O_12_	467.12	287.06 (9), 259.06 (80), 231.07 (56), 167.03 (22), 153.02 (86), 149.02 (69), 123.04 (26)	[30,31]
19	Quercetin diglucoside	8.7	C_27_H_30_O_17_	627.17	303.05 (100)	[30]
20	Quercetin malonyl Glc Glc	9.1	C_30_H_32_O_20_	713.16	465.10 (10), 303.05 (100)	
21	Cyanidin diglucoside	9.2	C_27_H_31_O_16_	611.16	287.06 (100)	[30]
22	Cyanidin rutinoside	9.5	C_27_H_31_O_15_	595.17	287.06 (100)	^2^
23	Cyanidin-3-Glc-5-(6-Mal)Glc	9.8	C_30_H_33_O_19_	697.16	449.11 (12), 287.06 (100)	[10]
24	Cyanidin glucoside	11.1	C_21_H_21_O_11_	449.11	287.06 (100)	[30]
25	Quercetin rutinoside	11.6	C_27_H_30_O_16_	611.16	303.05 (100)	[32]
26	Quercetin glucoside	12.0	C_21_H_20_O_12_	465.10	303.05 (100)	[30,32]
27	Anthocyanin A *	12.1	C_51_H_53_O_26_	1081.28	1081.28 (100), 919.23 (79), 449.11 (24), 287.06 (45)	[10,18]
28	Anthocyanin B (iso1) *	12.9	C_54_H_55_O_29_	1167.28	1167.28 (100), 1005.25 (11), 919.23 (8), 535.11 (15), 287.06 (19)	[10,18]
29	Anthocyanin B (iso2) *	13.4	C_54_H_55_O_29_	1167.28	1167.28 (100), 1005.23 (57), 449.11 (9), 287.06 (9)	[10,18]
30	Anthocyanin C *	13.5	C_51_H_53_O_25_	1065.29	1065.29 (99), 903.23 (96), 449.11 (28), 287.06 (62)	[10,18]
31	Naringenin glucoside	13.8	C_21_H_22_O_10_	435.13	273.08 (100), 153.02 (68), 147.04 (44)	[33]
32	Apigenin galacturonide	14.1	C_21_H_18_O_11_	447.09	271.06 (100)	[32]
33	Anthocyanin D (iso1) *	14.3	C_54_H_55_O_28_	1151.29	1151.29 (100), 903.23 (13), 535.11 (24), 287.06 (16)	[10,18]
34	Cyanidin malonylglucoside	14.8	C_24_H_30_O_14_	535.11	287.06 (100)	[10]
35	Anthocyanin D (iso2) *	14.9	C_54_H_55_O_28_	1151.29	1151.29 (100), 989.24 (68), 449.11 (11), 287.06 (29)	[10,18]

^1^ In brackets are reported the average relative abundances of each ion. ^2^ Identified by standard.* A: cyanidin-3-(6-*p*-coumaroyl-6’-caffeoyl)sophoroside-5-glucoside; * B (iso1): cyanidin-3-(6-*p*-coumaroyl-6’-caffeoyl)sophoroside-5-(6-malonyl)glucoside; *B (iso2): cyanidin-3-(6-*p*-coumaroyl-malonyl-6’-caffeoyl)sophoroside-5-glucoside; * C: cyanidin-3-(6,6’-di-*p*-coumaroyl)sophoroside-5-glucoside; * D (iso1): cyanidin-3-(6,6’-di-*p*-coumaroyl)sophoroside-5-(6-malonyl)glucoside; * D (iso2): cyanidin-3-(6-*p*-coumaroyl-malonyl-6’-*p*-coumaroyl)sophoroside-5-glucoside.
